# Ground-based measurements of the weather-driven sky radiance distribution in the Southern Hemisphere

**DOI:** 10.1371/journal.pone.0286397

**Published:** 2023-06-14

**Authors:** Raúl R. Cordero, Sarah Feron, Edgardo Sepúlveda, Alessandro Damiani, Jose Jorquera, Penny M. Rowe, Jorge Carrasco, Juan A. Rayas, Pedro Llanillo, Shelley MacDonell, Gunther Seckmeyer

**Affiliations:** 1 Universidad de Santiago de Chile, Santiago, Chile; 2 Knowledge Infrastructure, University of Groningen, Leeuwarden, Netherlands; 3 Center for Climate Change Adaptation, National Institute for Environmental Studies, Tsukuba, Japan; 4 NorthWest Research Associates, Redmond, WA, United States of America; 5 University of Magallanes, Punta Arenas, Chile; 6 Centro de Investigaciones en Óptica A. C., León, Gto, México; 7 Alfred Wegener Institute (AWI), Bremerhaven, Germany; 8 Centro de Estudios Avanzados en Zonas Áridas (CEAZA), La Serena, Chile; 9 Leibniz Universität Hannover, Hannover, Germany; Valahia University of Targoviste, ROMANIA

## Abstract

The angular distribution of the sky radiance determines the energy generation of solar power technologies as well as the ultraviolet (UV) doses delivered to the biosphere. The sky-diffuse radiance distribution depends on the wavelength, the solar elevation, and the atmospheric conditions. Here, we report on ground-based measurements of the all-sky radiance at three sites in the Southern Hemisphere across a transect of about 5,000 km: Santiago (33°S, a mid-latitude city of 6 million inhabitants with endemic poor air quality), King George Island (62°S, at the northern tip of the Antarctic Peninsula, one of the cloudiest regions on Earth), and Union Glacier (79°S, a snow-covered glacier in the vast interior of Western Antarctica). The sites were strategically selected for studying the influence of urban aerosols, frequent and thick clouds, and extremely high albedo on the sky-diffuse radiance distribution. Our results show that, due to changing site-specific atmospheric conditions, the characterization of the weather-driven sky radiance distribution may require ground-based measurements.

## 1. Introduction

The solar energy delivered to objects of complex shape (including buildings, plants, and animals) depends on the angular distribution of the radiance (i.e., the radiant flux per differential solid angle and surface element). By definition, the diffuse *irradiance* of a sloping plane can be calculated by integrating the *radiance* distribution of the sky ’seen’ by the plane [1]. Reliable information on the sky-diffuse radiance distribution enables better estimations of the power output of photovoltaic (PV) modules [2–5], the energy production potential of tilted surfaces and building façades [6, 7], and the ultraviolet (UV) doses on humans [8–10].

Measuring the radiance distribution requires sampling the downwelling solar radiance across zenith and azimuthal viewing angles. These measurements can be conducted sequentially using sky-scanners [11–15] or they can be conducted at once using non-scanning motionless multidirectional systems [16–18]. The latter are fitted with a set of multidirectional radiance input optics able to sample the radiance from several directions at the same time. For the visible part of the spectrum, simple and inexpensive variants of the multidirectional systems are based on all-sky cameras [19–21]. However, calibration of image or multidirectional sensors remains challenging [18].

The sky radiance distribution is strongly influenced by the same parameters (such as clouds, albedo, and aerosols) that determine the surface irradiance (i.e., the radiant flux per surface element). Clouds strongly modulate the angular distribution of the radiance. Under cloudless and unpolluted conditions, the downwelling sky-diffuse radiance is generally low close to the zenith. Except for moisture-rich optically thick clouds, the zenith radiance tends to increase under overcast conditions relative to cloudless conditions [12]. For high-albedo, snow-covered surfaces, the radiance is considerably intensified by multiple reflections between the surface and the scattering atmosphere [13, 14].

Despite these prior efforts, measurements in more diverse atmospheric conditions are needed. For example, although aerosols also affect the radiance, the effect of heavy urban pollution and/or dust on the angular distribution of the radiance distribution has not been measured. Additional measurements may also provide useful metrics for supporting the development of radiation models [22–24].

**Fig 1:**
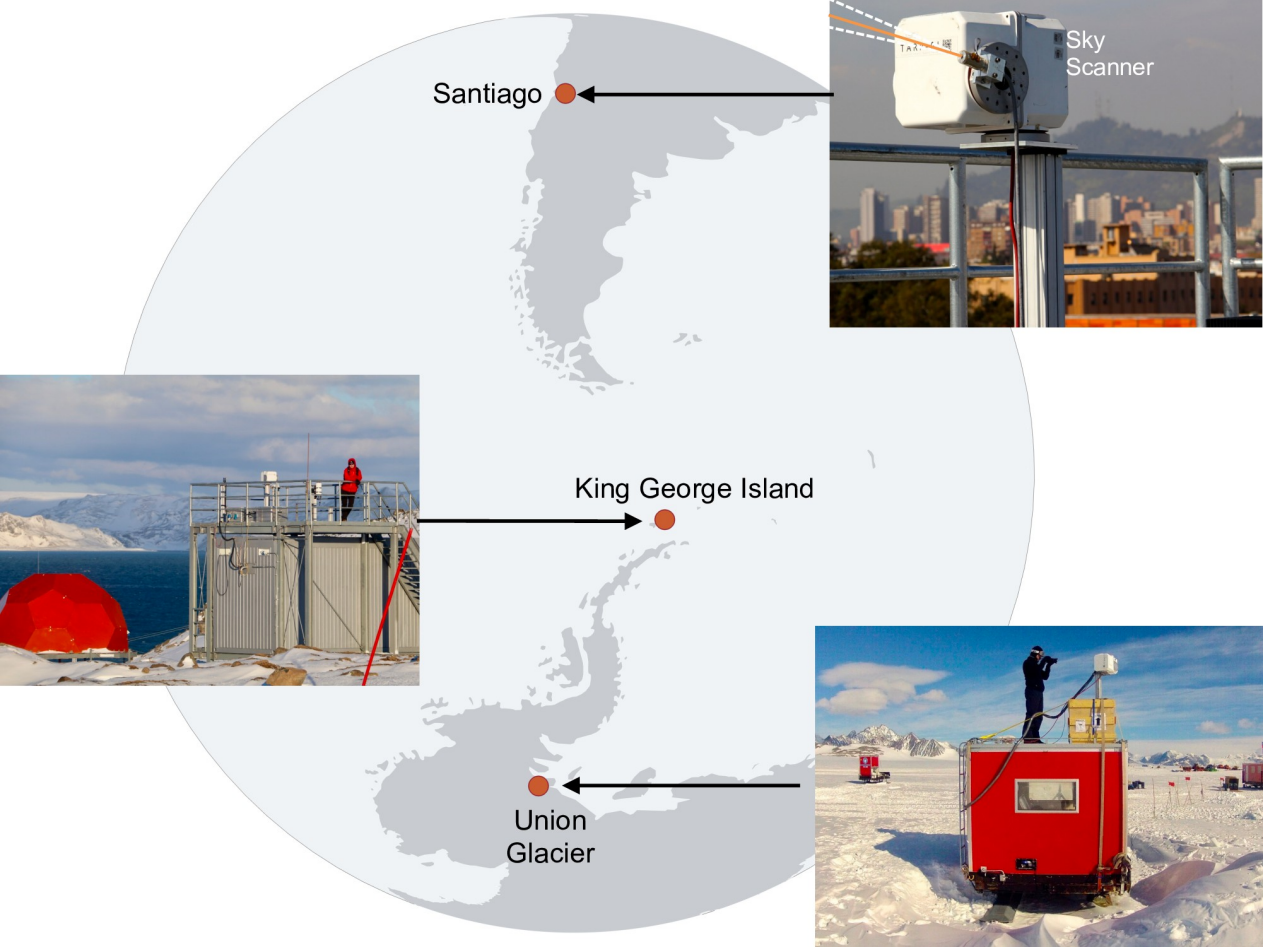
Measuring sites. The sky-scanner system that conducted the all-sky radiance measurements was programmed to sample the radiance at intervals of 15 degrees along both spherical coordinates (*θ* and *φ*) such that a total of 150 measurements resulted from each scan. A single-wavelength scan took roughly 8 minutes. In the case of downtown Santiago de Chile (33.4467°S, 70.6827°W), the gray sky in the picture is largely attributable to urban pollution. Thick clouds are frequent on King George Island (62.2013°S, 58.9658°W). The snow on Union Glacier (79.7669°S, 82.9144°W) is one of the cleanest on Earth, which makes the local albedo extremely high. Photographs taken by the authors. Plots and maps were generated by using Python’s Matplotlib Library [54].

Here, we report on ground-based measurements of the all-sky radiance distribution at three sites in the Southern Hemisphere (Fig 1 and S1-S3 Figs in S1 File) selected along a transect of about 5,000 km: Santiago (33°S, a polluted mid-latitude city of 6 million inhabitants), King George Island (62°S, at the northern tip of the Antarctic Peninsula, one of the cloudiest regions on Earth), and Union Glacier (79°S, a snow-covered glacier in the interior of Western Antarctica). A total of 652 measurements were conducted by a sky–scanner system [12, 14] to study the influence of urban aerosols (Santiago), thick clouds (King George Island), and high albedo (Union Glacier, Antarctica) on the sky radiance distribution.

## 2. Material and methods

### 2.1 Measuring sites

On King George Island, at the northern extreme of the Antarctic Peninsula, the surface radiation is driven by frequent and thick clouds. King George Island is the largest of the South Shetland Islands, lying 120 km off the coast of Antarctica in the Southern Ocean. The direct contribution (by scattering and absorption) of marine aerosol to the radiative budget is small in the predominantly pristine Antarctic. However, marine aerosols play an important role through cloud droplet activation, which determines cloud radiative properties [25]. Clouds on King George Island are more frequent and have a larger optical depth compared with other Antarctic sites [26]. While the cloud fraction (CF) is close to 1 according to ERA5 reanalysis dataset [27] (S4 Fig in S1 File), the cloud modification factor (CMF) for summer months (DJF) is about 0.5 in the ultraviolet and in the visible range of the spectrum according to satellite products [26]. ERA5 data are available at https://cds.climate.copernicus.eu/cdsapp#!/dataset/derived-near-surface-meteorological-variables?tab=overview. The CMF, often used for characterizing the clouds attenuation on the surface solar radiation, is the ratio between the measured all-sky surface radiation and the corresponding clear-sky surface radiation computed using a radiative transfer model. We conducted our measurements in early summer (December 2016) at the Chilean Escudero Station (62.2013°S, 58.9658°W) located in an ice-free area at Maxwell Bay, one of the three major bays on the island.

In Santiago, Chile (a mid-latitude city of 6 million inhabitants) the surface radiation is modulated by urban pollution and dust (resulting from the semi-arid climate). Aerosol dispersion is impeded by the complex topography surrounding Santiago and by a persistent regional subsidence thermal inversion layer that leads to stable stratified air [28]. The thermal inversion strengthens during fall and winter, increasing the trapping of pollutants and leading to multi-day episodes of high particulate matter concentrations [29]. The aerosol load was larger some decades ago before environmental policies began to be adopted in Santiago; driven by air-quality regulations, the daily fine (PM2.5) particle matter fraction has dropped by approximately 60% during the last two decades in Santiago [30]. Day-to-day changes in the aerosol optical depth (AOD) in Santiago are sensitive to variations in the strength of the subsidence thermal inversion and in the boundary-layer winds [28]. Prior measurements indicate that the AOD at 350 nm typically ranges from 0.2 to 0.3, with peak values of about 0.7 [28]. The AOD diminishes with wavelength and typically ranges from 0.1 to 0.2 at 550 nm [28]. Clouds play a relatively small role in the radiation climate of Santiago, especially in summer, when the CMF is higher than 0.9 [31]. We conducted our measurements at the campus of the University of Santiago (33.4467°S, 70.6827°W), located downtown Santiago, during two periods: summer (January) and winter (June 2019).

At Union Glacier, Antarctica, the surface radiation is strongly influenced by the high albedo of the perennially snow-covered surface, typical of the Antarctic interior (S5 Fig in S1 File). Union Glacier is a large glacier that receives the input from several tributaries and drains through the middle of the Heritage Range, Ellsworth Mountains. The glacier is the site of a blue-ice runway and two seasonally occupied camps (a Chilean camp and a camp operated by a private company that provides expedition support and tours). Despite these facilities, aerosol load is extremely low on Union Glacier due to strong katabatic winds that predominantly come from the southwest [32]. According to ERA5 reanalysis dataset [27], CF values on Union Glacier ranges from 0.3 to 0.4 for summer months (DJF). Snow albedo depends on snow characteristics (such as grain size), the morphology of the surface and the surroundings as well as the content of light-absorbing impurities [33, 34]. Ground-based measurements of the albedo at Union Glacier have shown that the albedo in the UV and the visible part of the spectrum is slightly higher than 0.9 [32]. We conducted our measurements at the Chilean Union Glacier Camp (79.7669°S, 82.9144°W), during two early summer campaigns (December 2012 [14] and December 2014).

### 2.2 Sky-scanner system

Our radiance measurements were carried out using a robotic sky scanner (see S1-S3 Figs in S1 File) developed from a customized sun tracker INTRA® with gears 14:1 (maximum speed 8.5 degrees per second). The unit was programmed to sample the radiance at intervals of 15 degrees along both spherical coordinates (*θ* and *φ*) such that a total of 150 measurements resulted from each scan. Note that our 150-points selection covers a smaller fraction of the sky hemisphere than the Tregenza-based systems [15], which results in a lower spatial resolution. Although we could program additional sampling points for matching the spatial resolution of Tregenza-based systems, this would increase the scanning time lowering the temporal resolution of the system and making it harder to track the effects of fast-moving clouds, for example. Thus, our 150-points selection appears to be a good compromise between spatial resolution and temporal resolution.

The input optics consists of a 130 mm long collimator tube. A baffle is used to limit the field of view (FOV) and to avoid stray light. The FOV is approximately 4.5 degrees, which corresponds to a solid angle of 4.8 x 10^−3^ steradian (sr) [12]. Note that our measurements of the horizon radiance, partially capture the radiation reflected by the surface. The influence of the surface may induce a slight low bias in our measurements of the horizon radiance, especially at locations where the albedo is relatively low (i.e., Santiago). A thinner FOV may allow us to slightly reduce such influence but, considering the limited sampling points (150), it would also reduce our spatial resolution making it harder to capture some spatial features of the radiance under broken cloud conditions, for example. Nevertheless, we tested this effect in prior efforts [12] comparing measurements of the horizon radiance with model simulations; we found differences within the uncertainty bounds, which suggest that the surface-related bias is not considerable for the adopted FOV.

The radiance was sampled using a double monochromator-based spectroradiometer (Bentham DMc150F-U) with a 150mm focal length and 1800 lines/mm gratings. The system is operated within a weatherproof box fitted with an active temperature-controlling system. The Full Width at Half Maximum (FWHM) of the spectroradiometer is 0.8 nm (in the range 300–800 nm). The instrument complies with the specifications of the Network for the Detection of Atmospheric Composition Change (NDACC) [35] and the World Meteorological Organization (WMO) recommendations [36] for measurements in the UV part of the spectrum. Instead of commercial systems (e.g., EKO sky scanners or PRC Krochmann) that are mostly focused on broadband measurements (i.e., luminance), our system enables spectrally-resolved measurements.

Synchronization of the sky scanner and the spectroradiometer was achieved using a software program in NI LabView [37]. Sampling the radiance at each point in the sky took about 1 s and, operating at maximum speed, the sky-scanner took about 2 s to move the input optics to the next consecutive point in the sky. Accordingly, a single-wavelength scan of the entire sky (150 measurements) took roughly eight minutes. Note that changing atmospheric conditions (fast moving clouds in particular) may have affected our measurements during the roughly eight minutes that took us to complete a sky scan. This limitation can only be fully overcome by non-scanning motionless multidirectional systems [16–18].

In our case, the absolute calibration of the system was achieved by using a 200-mm diameter integrating sphere fitted with a baffled 50-W quartz halogen lamp. Based on the calibration certificate of the lamp, the standard uncertainty of our measurements is estimated to be about 5% for wavelengths longer than 320 nm [38, 39].

**Fig 2:**
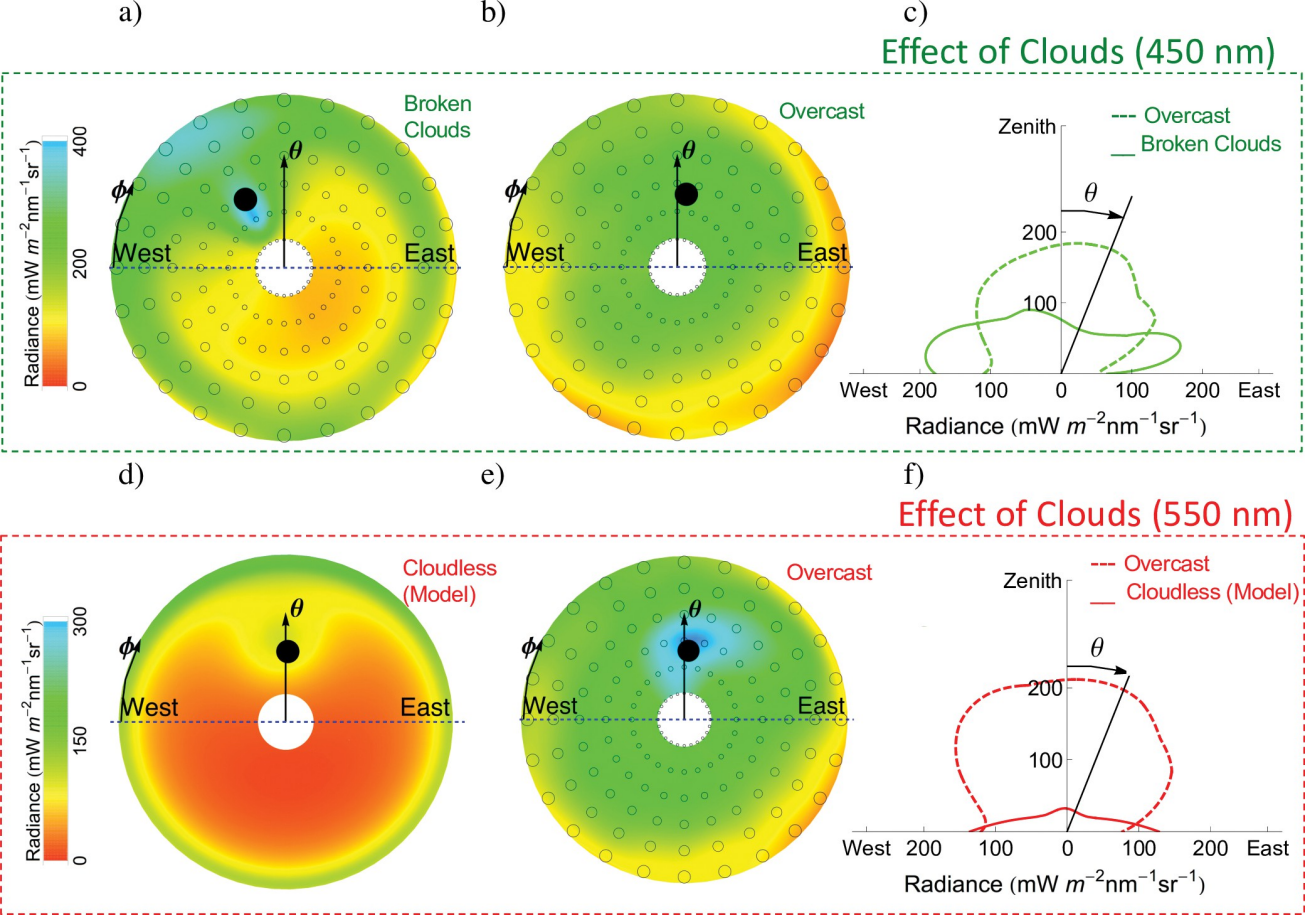
Radiance distribution on King George Island (early summer). Frequent clouds make the zenith radiance larger (relative to cloudless conditions). a) Radiance distribution (450 nm) measured under broken cloud conditions on 9 Dec 2016. **Circles indicate the measuring points except for the black filled-in circle that stands for the sun position.** The solar zenith angle (SZA) and solar azimuth angle (SAA) at the moment of the measurements were 40° and 61°, respectively; b) Radiance distribution (450 nm; SZA = 39°; SAA = 97°; 9 Dec 2016) measured under overcast conditions; c) Angular distribution of the radiance in the plane “West-Zenith-East”. **This plot shows the radiance measured changing the zenith angle (*θ*) while keeping the azimuth angle (*ϕ*) constant at 0°.** Radiance data were obtained from plots a) and b). If the radiance were perfectly isotropic, this plot would show semicircles; d) Radiance distribution (550 nm; SZA = 39°; SAA = 91°) computed by using the UVSPEC model assuming cloudless conditions; e) Radiance distribution (550 nm; SZA = 39°; SAA = 91°; 10 Dec 2016) measured under overcast conditions; f) Angular distribution of the radiance in the plane “West-Zenith-East”. Radiance data were obtained from plots d) and e). **Coordinates *x* and *y* in plots c) and f) were computed considering that the radiance (*r*) and the zenith angle (*θ*) are polar coordinates: *x* = *r*.cos*θ y* = *r*.sin*θ*.** Plots were generated by using Python’s Matplotlib Library [54].

The origin of the spherical coordinate system (θ = 0°; φ = 0°) is indicated in Fig 2A. If the radiance were perfectly isotropic, this ratio would be equal to 1.

### 2.3 Radiative transfer model

Additional comparisons involved estimates rendered by the UVSPEC radiative transfer model [40]. In the case of the solar irradiance, the model has been validated via systematic comparisons with ground-based measurements under cloudless conditions [41, 42]. The model uses the DIScrete Ordinates Radiative Transfer (DISORT) solver [43] and the extraterrestrial spectrum by Gueymard [44]. The model allows modification of standard atmospheres provided by Anderson et al. [45]; in particular, the user can modify aerosols and cloud properties as well as the total column of ozone and precipitable water. In the cases of Santiago and King George Island, local measurements of the aerosol optical depth (AOD) carried out by AERONET-affiliated photometers were used [46], while in the case of Union Glacier an AOD of 0.02 was used (assumed to be appropriate for the extremely low aerosol load in the Antarctic interior).

Under cloudless conditions, the standard uncertainty associated with UVSPEC irradiance estimates in the visible range has been found to be strongly influenced by the aerosol load, ranging from about 5% under low aerosol conditions, up to about 15% for polluted air [47, 48]. In the case of radiance, under cloudless conditions, differences between model outcomes and ground-based measurements have been found to be within the ±20% bounds in the visible range [12, 14]. Considering the limitation of the UVSPEC model in the case of cloudy conditions, here we applied the UVSPEC model to simulate the radiance distribution under cloudless conditions.

## 3. Results

Clouds generally enhance the zenith radiance (relative to cloudless conditions). Our measurements of sky radiance distributions on King George Island, one of the cloudiest sites on Earth, aimed at better understanding the effect of clouds. Fig 2 (first row) enables the comparison of radiance distributions measured under different cloud conditions. The radiance at the horizon is generally larger than the zenith radiance under broken cloud conditions (Fig 2A), while the reverse is true for overcast skies (Fig 2B). The contrast between the zenith radiance and the radiance at the horizon is even greater when comparing clear skies to overcast skies; plots in Fig 2 (second row) compare the radiance distribution measured under overcast conditions with the radiance distribution computed for King George Island assuming cloudless conditions.

Despite the wavelength and solar elevation (or the time of the day), S7 Fig in S1 File shows that the zenith radiance increases under overcast conditions (relative to cloudless conditions) on King George Island. Clouds in this region, at the northern tip of the Antarctic Peninsula, are more frequent and have a larger optical depth compared with other Antarctic sites [26]. S6 Fig in S1 File shows a set of whole-sky pictures taken by a fisheye cloud camera at the site on King George Island where we conducted our measurements.

The ratio between the radiance at the horizon (*θ* = 90°; *φ =* 180°) and the zenith radiance (*θ* = 0°) changes throughout the day at King George Island according to the changing cloud conditions. Under cloudless conditions, model simulations indicate that the ratio would range throughout the day from 1 to 50 (mean 7) at 550 nm, and from 2 to 10 (mean 5) at 450 nm. However, under the frequent and thick clouds characteristic of King George Island (S4 Fig in S1 File), the larger zenith radiance causes the ratio to be considerably closer to 1 throughout the day.

**Fig 3:**
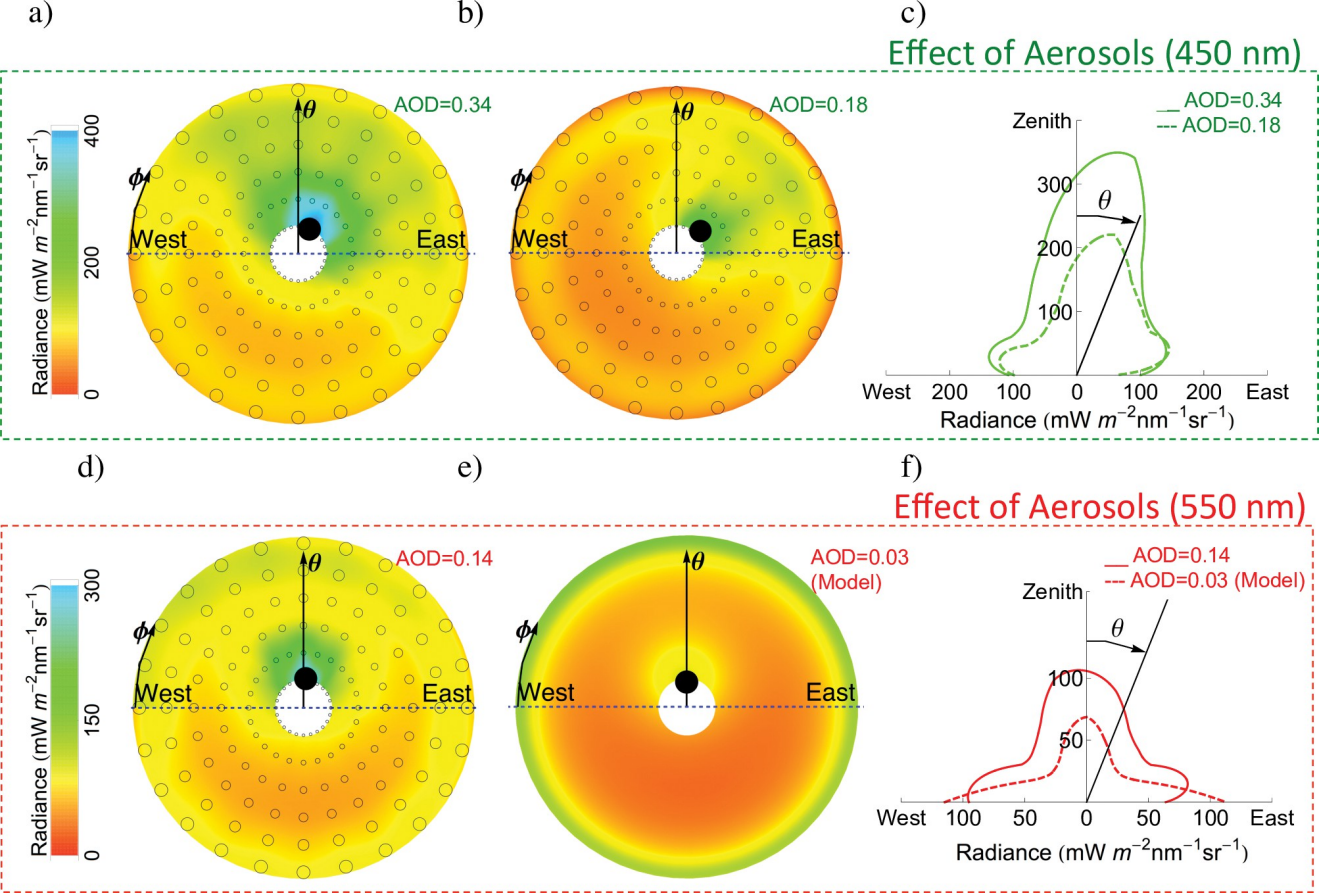
Radiance distribution in Santiago (summer). **Urban pollution makes zenith radiance larger (relative to unpolluted conditions).** a) Radiance distribution (450 nm; SZA = 15°; SAA = 105°; 20 Jan 2015) measured under cloudless conditions when the AOD was 0.34. Circles indicate the measuring points except for the black filled-in circle that stands for the sun position; b) Radiance distribution (450 nm; SZA = 21°; SAA = 135°; 22 Jan 2015) measured under cloudless conditions when the AOD was 0.18; c) Angular distribution of the radiance in the plane “West-Zenith-East”. This plot shows the radiance measured changing the zenith angle (*θ*) while keeping the azimuth angle (*ϕ*) constant at 0°. Radiance data were obtained from plots a) and b). If the radiance were perfectly isotropic, this plot would show semicircles; d) Radiance distribution (550 nm; SZA = 18°; SAA = 91°; 22 Jan 2015) measured under cloudless conditions when the AOD was 0.14; e) Radiance distribution (550 nm; SZA = 18°; SAA = 90°) computed using the UVSPEC model assuming unpolluted conditions (AOD = 0.03); f) Angular distribution of the radiance in the plane “West-Zenith-East”. Radiance data were obtained from plots d) and e). **Coordinates *x* and *y* in plots c) and f) were computed considering that the radiance (*r*) and the zenith angle (*θ*) are polar coordinates: *x* = *r*.cos*θ y* = *r*.sin*θ***. Plots were generated by using Python’s Matplotlib Library [54].

Similar to thin clouds, aerosols (anthropogenic particle material and dust) also enhance the zenith radiance (relative to unpolluted conditions). Our measurements of sky radiance distributions in Santiago de Chile (Fig 3) show the strong effect on the zenith radiance of persistent aerosols, which include urban pollution and dust (resulting from the semi-arid climate in the Santiago region).

Fig 3 (first row) compares radiance distributions measured under different aerosol loadings; the measurements were conducted on consecutive days such that the solar zenith angle (SZA) and the solar azimuth angle (SAA) at the moment of the measurement were nearly the same. As shown in Fig 3 (first row), the zenith radiance considerably increases with the aerosol optical depth (AOD). Without the aerosols, the zenith radiance in Santiago would be considerably less intense, which agrees with recent efforts aimed at modeling the radiance distribution under variable aerosol conditions [22]. Plots in Fig 3 (second row) compare the radiance distribution measured in Santiago with the radiance distribution computed for Santiago assuming clean conditions (we assumed an AOD equal to 0.03).

There are considerable seasonal changes in the radiance distribution; the sun is much closer to the zenith in summer than in winter, which makes the zenith radiance larger close to noon. In high-latitude regions like Antarctica, the sun is absent for much of the year (making measurements irrelevant). In contrast, at mid-latitude locations like Santiago measurements conducted in winter (S8 Fig in S1 File) are pertinent and provide information for assessing the effect of seasonal changes in the solar elevation. The zenith radiance drops considerably in winter (relative to the summer values; compare S8 Fig in S1 File and Fig 3). The wintertime drop in the zenith radiance makes the ratio between the horizon radiance (*θ* = 90°; *φ =* 180°) and the zenith radiance (*θ* = 0°) slightly higher in winter than in summer for Santiago.

**Fig 4:**
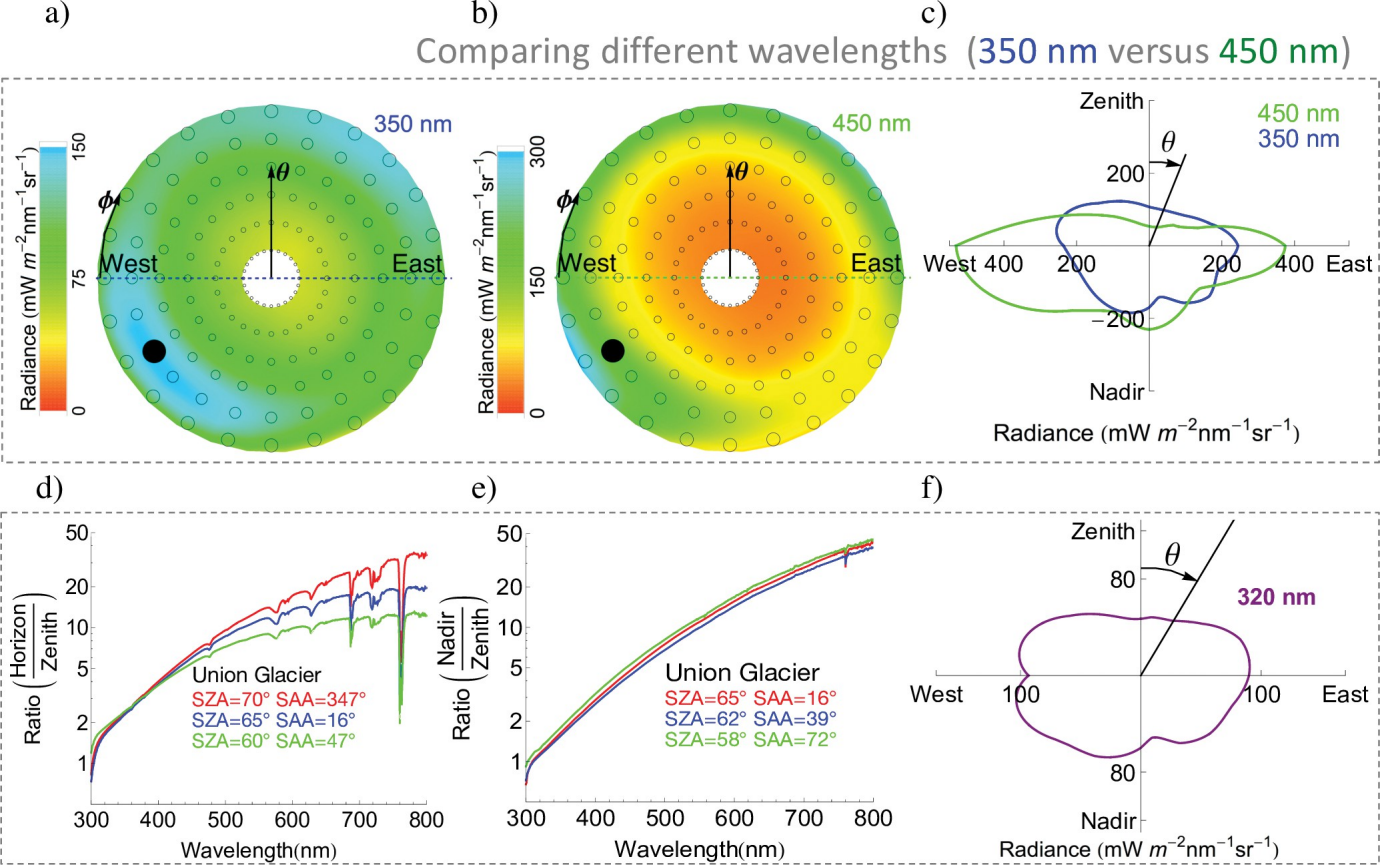
Radiance distribution on Union Glacier (summer). a) Radiance distribution (350 nm; SZA = 75°; SAA = 330°; 2 Dec 2014) measured under cloudless conditions. Circles indicate the measuring points except for the black filled-in circle that stands for the sun position; b) Radiance distribution (450 nm; SZA = 75°; SAA = 330°; 2 Dec 2014) measured under cloudless conditions; c) Angular distribution of the radiance in the plane “West-Zenith-East-Nadir” conducted when the SZA was 62° (350 nm, see blue line) and 67° (450 nm, see red line). This plot shows the radiance measured changing the zenith angle (*θ*) while keeping the azimuth angle (*ϕ*) constant at 0°. If the radiances were perfectly isotropic, this plot would show circles. d) Ratio between the horizon radiance (*θ* = 90°; *φ =* 180°) and the zenith radiance (*θ* = 0°) measured on Union Glacier; e) Ratio between the nadir radiance (*θ* = 180°) and the zenith radiance (*θ* = 0°) measured on Union Glacier; f) Angular distribution of the radiance in the plane “West-Zenith-East-Nadir” conducted on Union Glacier (early summer) when the SZA was 66°. **Plots d) and e) are based on spectral measurements shown in the supporting information (S10 Fig in S1 File).** The solar zenith angle (SZA) and the solar azimuth angle (SAA) at the moment of the measurement are indicated in the plots. **Coordinates *x* and *y* in plots c) and f) were computed considering that the radiance (*r*) and the zenith angle (*θ*) are polar coordinates: *x* = *r*.cos*θ y* = *r*.sin*θ*.** Plots were generated by using Python’s Matplotlib Library [54].

The high albedo of snow-covered surfaces makes the downwelling radiance more intense. Our measurements of radiance distributions on Union Glacier (Fig 4) enhanced our understanding of the effect of the albedo on both the downwelling and the upwelling radiance distributions. Note that Fig 4C includes measurements of the upwelling radiance in the plane “West-Zenith-East-Nadir” that were conducted by pointing the input optics at the surface. If the radiance were perfectly isotropic, Fig 4C would show circles.

Attributable to the multiple reflections between the surface and the scattering atmosphere (i.e., backscattering effect) [32], the radiance was found to increase with the surface albedo at all wavelengths and for all viewing angles (S9 Fig in S1 File). However, the influence of the reflecting surface tends to be larger for viewing angles close to the horizon (i.e., close to the reflecting surface). According to model simulations, the ratio between the horizon radiance (*θ* = 90°; *φ =* 180°) and the zenith radiance (*θ* = 0°) is larger over snow-covered surfaces than over dark surfaces (S9c Fig in S1 File). The effect of the albedo on the horizon radiance combined with the predominantly cloudless conditions and the aerosol scarcity makes the downwelling radiance distribution on Union Glacier persistently anisotropic beyond the UV part of the spectrum. The persistently high horizon radiance over snow/covered surfaces in the visible part of the spectrum affects the power output of solar PV modules and should be taken into account when deciding the ideal angle for solar panel installations over highly reflecting surfaces. Yet, note that in the case of large (multi-row) utility-scale systems, potential beneficial effect of vertically setting PV modules almost completely vanishes because of the modules’ self-shading.

As shown in Fig 4D, in the infrared part of the spectrum (wavelengths longer than 700 nm), the horizon radiances (*θ* = 90°; *φ =* 180°) can be ten or more times larger than the zenith radiance (*θ* = 0°). Interestingly, a number of absorption bands of atmospheric gases are apparent in Fig 4D, including, for example, O_2_ absorption bands (around 627, 687 and 759 nm). These absorption bands show that the radiance at the horizon is undergoing (at some specific wavelengths) a stronger absorption than the zenith radiance. The stronger absorption results from the different optical mass through the atmosphere; the optical mass is shorter for the zenith radiance than for the horizon radiance. Similarly, MAX-DOAS instruments use the radiance measured at different elevation angles to detect the presence of certain gases [49, 50].

The nadir radiance also increases with the surface albedo for all wavelengths. The angular distribution of the upwelling radiance from snow-covered surfaces is generally more isotropic than the downwelling radiance [14]. This makes the nadir radiance generally larger than the zenith radiance. As shown in Fig 4E, the nadir radiance (*θ* = 180°) can also be dozens of times larger than the zenith radiance (*θ* = 0°) for wavelengths longer than 700 nm. The difference between the nadir radiance and zenith radiance is considerably smaller in the UV part of the spectrum and the ratio between the horizon radiance (*θ* = 90°; *φ =* 180°) and the zenith radiance (*θ* = 0°) as well as the ratio between the nadir radiance (*θ* = 180°) and the zenith radiance (*θ* = 0°) becomes closer to 1 (see Fig 4D and 4E). Accordingly, our measurements show that the angular distribution of the radiance is considerably more isotropic at 320 nm (Fig 4F) than at 450 nm (Fig 4C). This result was expected since shorter wavelengths are scattered more than longer wavelengths, which in turn makes the radiance distribution more isotropic in the UV range than in the visible part of the spectrum.

As supporting information (S11 Fig in S1 File), we have included boxplots of the ratio between the horizon radiance (*θ* = 90°; *φ =* 180°) and the zenith radiance (*θ* = 0°) computed from the total of scans analyzed in this research. The dispersion of values (the size of the boxes) shown in S11 Fig in S1 File is attributable to changes in both the solar position and the atmospheric conditions (clouds and aerosols). The distinctive patterns of the boxplots suggest that features of the radiance distributions corresponding to a specific site cannot always be extrapolated to other sites, which in turn underlines the need for site-specific assessments. The relatively small dispersion indicated by the boxes corresponding to the measurements on Union Glacier are mostly attributable to changes in the solar position since the atmospheric conditions exhibit few changes during summer on Union Glacier (aerosol load is extremely low and cloudless conditions are predominant). The frequent and thick clouds on King George Island boost the zenith radiance making the ratio between the horizon radiance (*θ* = 90°; *φ =* 180°) and the zenith radiance (*θ* = 0°) typically well below 1 even in the visible range (550 nm) of the spectrum. This ratio computed for 550 nm is generally larger than 1 under the predominantly cloudless conditions observed on Union Glacier and in Santiago (winter). The high solar elevation in Santiago brings the ratio between the horizon radiance (*θ* = 90°; *φ =* 180°) and the zenith radiance (*θ* = 0°) close to 1 in summer.

## 4. Discussion

The solar energy delivered to non-horizontal surfaces depends on the angular distribution of the radiance. Here, we report on ground-based measurements of the all-sky radiance at three sites in the Southern Hemisphere strategically selected for studying the influence of urban aerosols (Santiago, Chile), thick clouds (King George Island, Antarctica), and high surface albedo (Union Glacier, Antarctica) on the sky radiance distribution.

In keeping with prior work, we found that clouds enhance the zenith radiance relative to cloudless conditions (although as shown by Davis & Marshak thick clouds reduce the zenith radiance relative to thinner clouds [51]). The horizon radiance tends to be larger than the zenith radiance under both cloudless and broken cloud conditions. However, frequent and thick clouds make zenith radiance considerably larger bringing the ratio between the horizon radiance and the zenith radiance closer to 1.

Aerosols (anthropogenic particle material and dust) also enhance the zenith radiance (relative to unpolluted conditions). The latter considerably increases with the aerosol optical depth, which makes the ratio between the horizon radiance and the zenith radiance often close to 1 in polluted sites like Santiago. The seasonal changes in the solar elevations also affect the radiance distribution; since the sun is much closer to the zenith in summer than in winter, the zenith radiance considerably drops in winter (relative to the summer values). The wintertime drop in the zenith radiance makes the ratio between the horizon radiance and the zenith radiance slightly higher in winter than in summer in Santiago.

The high albedo of snow-covered surfaces considerably enhances the horizon radiance, (relative to the lower albedo of darker surfaces). The effect of the albedo on the horizon radiance combined with the predominantly cloudless conditions and the aerosols scarcity makes the horizon radiance on Union Glacier considerably larger than the zenith radiance. especially beyond the UV part of the spectrum.

We found that the nadir radiance also increases with the albedo, regardless of wavelength. The nadir radiance can be dozens of times larger than the zenith radiance in the visible part of the spectrum. However, the differences between the nadir radiance and zenith radiance as well as the differences between the horizon radiance and zenith radiance are considerably smaller in the UV part of the spectrum.

Our measurements also confirm that the angular distribution of the upwelling radiance from snow-covered surfaces is generally more isotropic than the downwelling radiance. This is useful information for modeling the power output of bifacial solar PV modules [52, 53]. These transparent-backsheeted modules expose their front and backside to the downwelling and the upwelling irradiance, respectively, increasing the energy yield per square meter of PV module.

We found that the sky-diffuse radiance distribution is strongly weather dependent and change with the solar position, the aerosol load, the surface albedo, and the distribution and optical properties of clouds. However, these parameters change over ranges constrained by local conditions. This is why the ratio between the horizon radiance and the zenith radiance, measured at different locations, exhibits such distinctive patterns (S11 Fig in S1 File). Our results confirm that features of the radiance distributions corresponding to a specific site can hardly be extrapolated to others.

Although we have produced the largest dataset of radiance observations available for South America and for Antarctica, the number of sites considered in this study was limited. Thus, our results alone cannot provide a full characterization of the radiance climatology in the southern hemisphere. Nevertheless, our measurements have revealed some interesting features regarding the weather-driven variability of the atmospheric transmittance in South America and Antarctica. Although the radiance distribution determines the power output of PV modules and the UV doses delivered to the biosphere, applications of this type of measurements still depend on developments beyond atmospheric sciences. Yet, we expect that our freely available set of ground-based measurements will provide useful insights for supporting the development of applications (related to solar energy or architecture, for example), the characterization of affordable instruments (focused on broadband measurements, for example), as well as the validation of existing radiative models such as Monte Carlo solvers (e.g., MYSTIC) [23] or Spherical Harmoic Discrete Ordinate Method (SHDOM) [24]. Our radiance measurements are available at https://figshare.com/articles/dataset/Radiance_Measurements/21498681.

## 5. Conclusions

The solar energy delivered to objects of complex shape (including buildings, plants, and animals) is determined by the angular distribution of the sky radiance, which is in turn strongly modulated by atmospheric conditions. Our ground-based measurements, conducted at three strategically selected sites in the Southern Hemisphere (from latitude 33°S to latitude 79°S) have shown that, while highly reflective surfaces considerably increase the horizon radiance (relative to darker surfaces), clouds and aerosols enhance the zenith radiance (relative to cloudless and unpolluted conditions). Although our measurements revealed some interesting features of the radiance climatology under different atmospheric conditions, they also confirmed that the sky-diffuse radiance distribution is strongly weather dependent and changes with the aerosol load, the surface albedo, and the distribution and optical properties of clouds. Since site-specific atmospheric conditions (i.e., aerosols, albedo, clouds) can hardly be extrapolated, we conclude that the characterization of the weather-driven sky radiance distribution may require ground-based measurements similar to those reported in this study.

## Supporting information

S1 File (PDF)
