# Supplementary material for: Ground-based measurements of the weather-driven sky radiance distribution in the Southern Hemisphere
Source: PLoS One. 2023 Jun 14;18(6):e0286397. doi: 10.1371/journal.pone.0286397 (PMC10266621; doi:10.1371/journal.pone.0286397)
Supplement: S1 File — (PDF) [file pone.0286397.s001.pdf]

Supporting Information

Ground-based measurements of the weather-driven sky radiance distribution in the Southern Hemisphere

Raúl R. Cordero[1], Sarah Feron[1,2*], Edgardo Sepúlveda[1], Alessandro Damiani[3], Jose Jorquera[1], Penny M. Rowe[4], Jorge Carrasco[5], Juan A. Rayas[6], Pedro Llanillo[7], Shelley MacDonell[8], Gunther Seckmeyer[9]

1 Universidad de Santiago de Chile. Av. Bernardo O'Higgins 3363, Santiago, Chile.
2 Knowledge Infrastructure, University of Groningen, Wirdumerdijk 34, 8911 CE Leeuwarden, Netherlands.
3 Center for Climate Change Adaptation, National Institute for Environmental Studies, Tsukuba 305-8506, Japan
4 NorthWest Research Associates, Redmond, WA, USA
5 University of Magallanes, Av. Manuel Bulnes 1855, Punta Arenas, Chile.
6 Centro de Investigaciones en Óptica A. C., Loma del Bosque 115 C. P. 37150, León, Gto. México
7 Alfred Wegener Institute (AWI), Am Handelshafen 12, 27570 Bremerhaven, Germany
8 Centro de Estudios Avanzados en Zonas Áridas (CEAZA), La Serena, Chile
9 Leibniz Universität Hannover, Herrenhauser Strasse 2, Hannover, Germany

* Corresponding Author
Sarah Feron
Wirdumerdijk 34, 8911 CE Leeuwarden,
8911 CE Leeuwarden, Netherlands
+49 1705967197

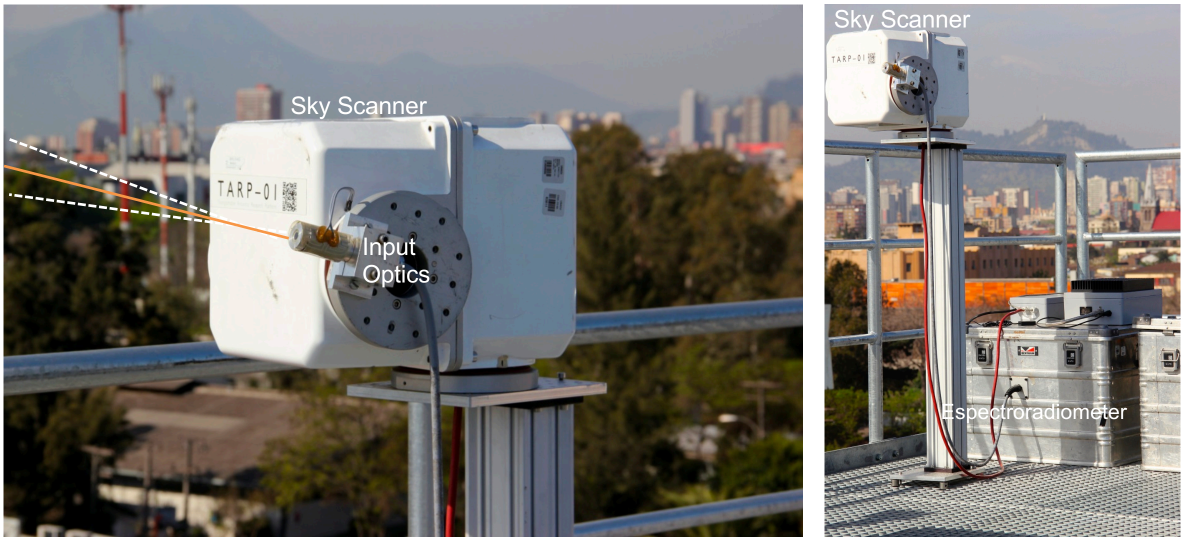

Fig. S1
Measurements of the angular distribution of the radiance (downtown Santiago de Chile, 33.4467ºS, 70.6827ºW). The gray sky is largely attributable to urban pollution. Photographs taken by the authors.

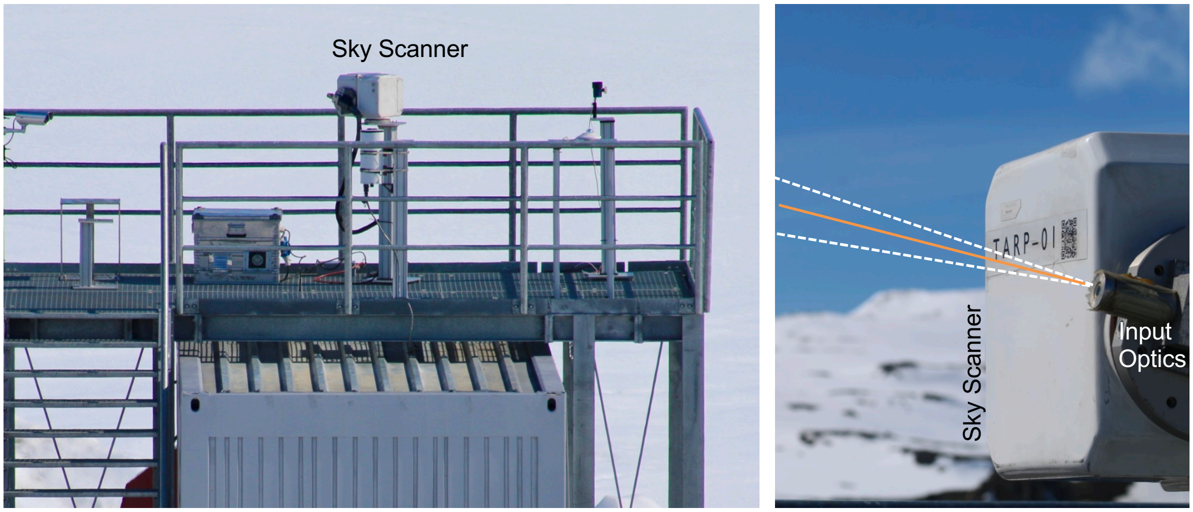

Fig. S2
Measurements of the angular distribution of the radiance on King George Island (62.2013ºS, 58.9658ºW; Antarctic Peninsula). Although the pictures were taken on a sunny day, the region is one of the cloudiest on Earth. Photographs taken by the authors.

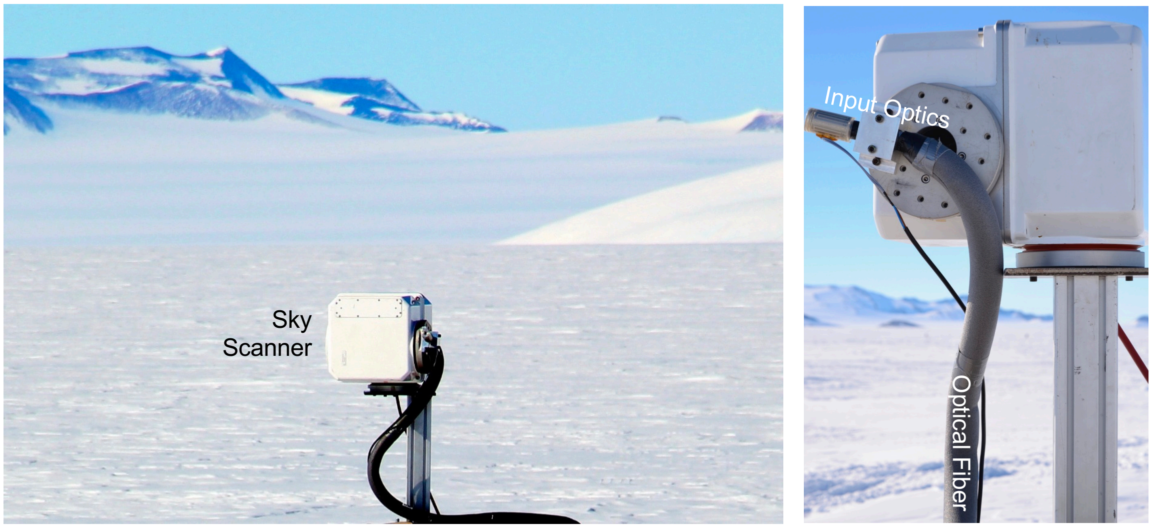

Fig. S3
Measurements of the angular distribution of the radiance on Union Glacier Camp (79.7669ºS, 82.9144ºW; West Antarctica). The snow at this deep-field Antarctica site is one of the cleanest on Earth, which makes the local albedo extremely high. Note the thermal isolation used for protecting the optical fiber. Photographs taken by the authors.

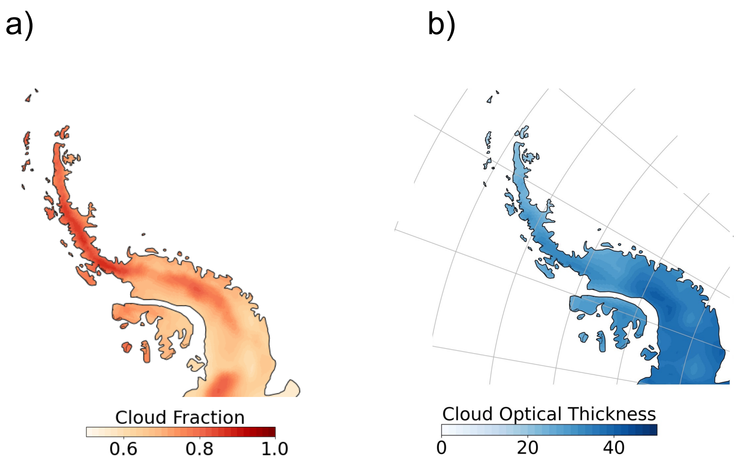

Fig. S4

a) Cloud fraction (*CF*) averaged for summer months (DJF) over the period 1981-2019. Data from ERA5 reanalysis [1] were used.

b) Cloud optical thickness averaged for summer months (DJF) over the period 2003-2019. Data from MODIS [2] were used.

Plot was generated by using Python's Matplotlib Library [3].

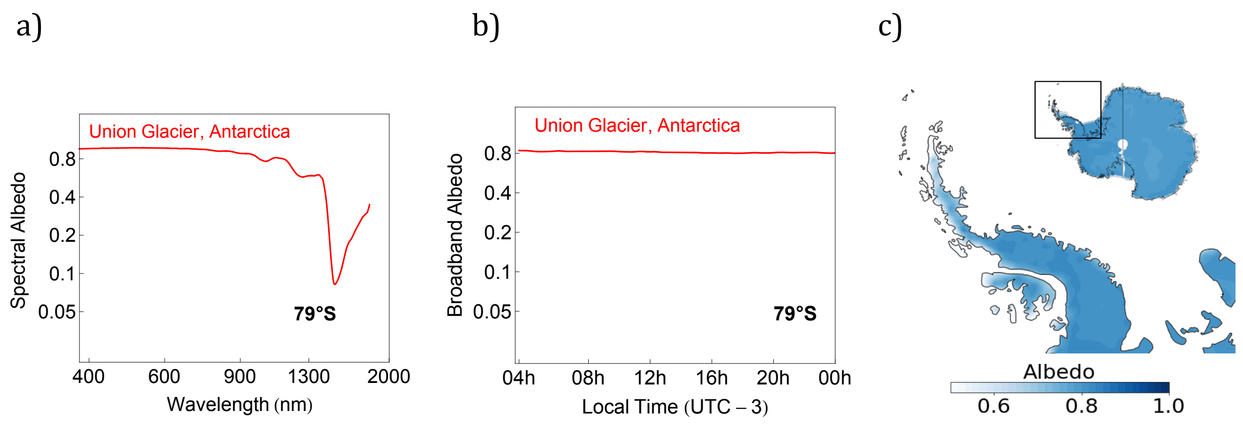

Fig. S5

Ground-based measurements of the albedo on Union Glacier (early summer).

a) Spectral albedo measured close to noon;
b) Broadband albedo measured during the day.
c) Broadband shortwave albedo averaged for December, January and February (DJF) days over the period 2004–2020. Data from MERRA-2 [4] were used.

Plots and maps were generated by using Python's Matplotlib Library [3].

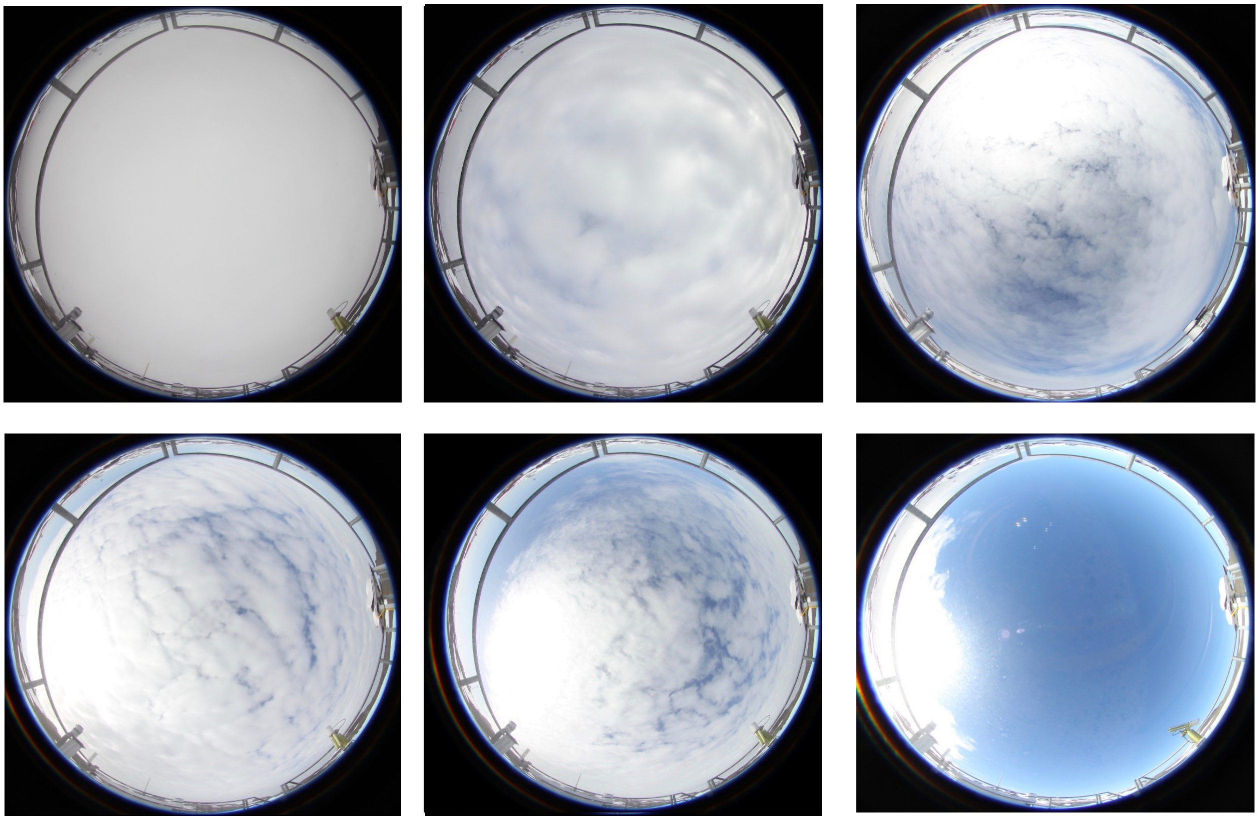

Fig. S6

Whole-sky pictures by a fisheye cloud camera taken in early summer on King George Island (62ºS, at the northern tip of the Antarctic Peninsula, one of the cloudiest regions on Earth). Clouds on King George Island are more frequent and have a larger optical depth compared with other Antarctic sites [5].

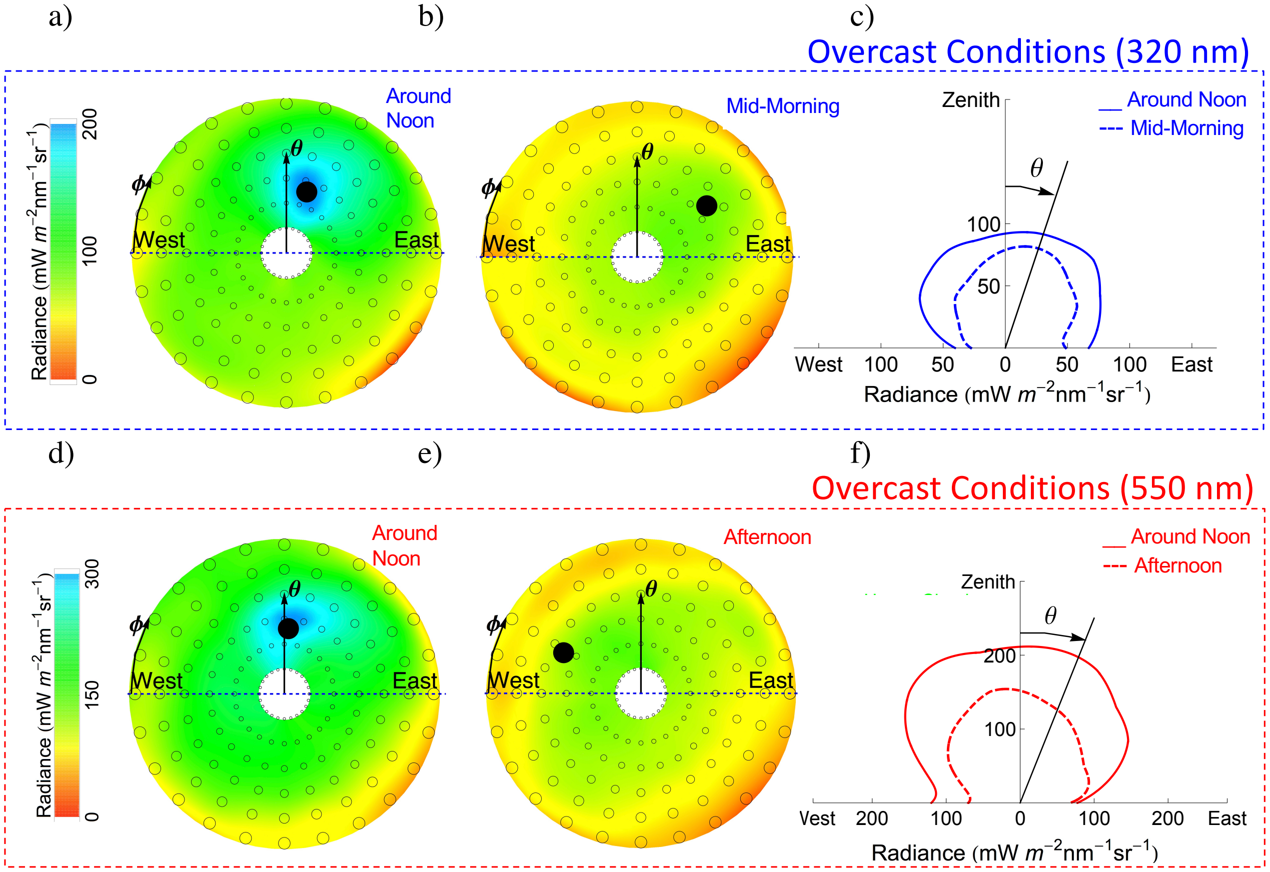

Fig. S7

Radiance distribution on King George Island (early summer). The zenith radiance increases under overcast conditions (relative to cloudless conditions), regardless of the wavelength and the solar elevation.

a) Radiance distribution (320 nm) measured around noon under overcast conditions. Circles indicate the measuring points except for the black filled-in circle that stands for the sun position;

b) Radiance distribution (320 nm) measured by midmorning under overcast conditions;

c) Angular distribution of the radiance in the plane "West-Zenith-East". This plot shows the radiance measured changing the zenith angle (θ) while keeping the azimuth angle (ϕ) constant at 0º. Radiance data were obtained from plots a) and b). If the radiance were perfectly isotropic, this plot would show semicircles;

d) Radiance distribution (550 nm) measured around noon under overcast conditions;

e) Radiance distribution (550 nm) measured afternoon under overcast conditions;

f) Angular distribution of the radiance in the plane "West-Zenith-East". Radiance data were obtained from plots d) and e).

Coordinates *x* and *y* in plots c) and f) were computed considering that the radiance (*r*) and the zenith angle (*θ*) are polar coordinates: $x=r.\cos\theta$ $y=r.\sin\theta$. Plots were generated by using Python's Matplotlib Library [3].

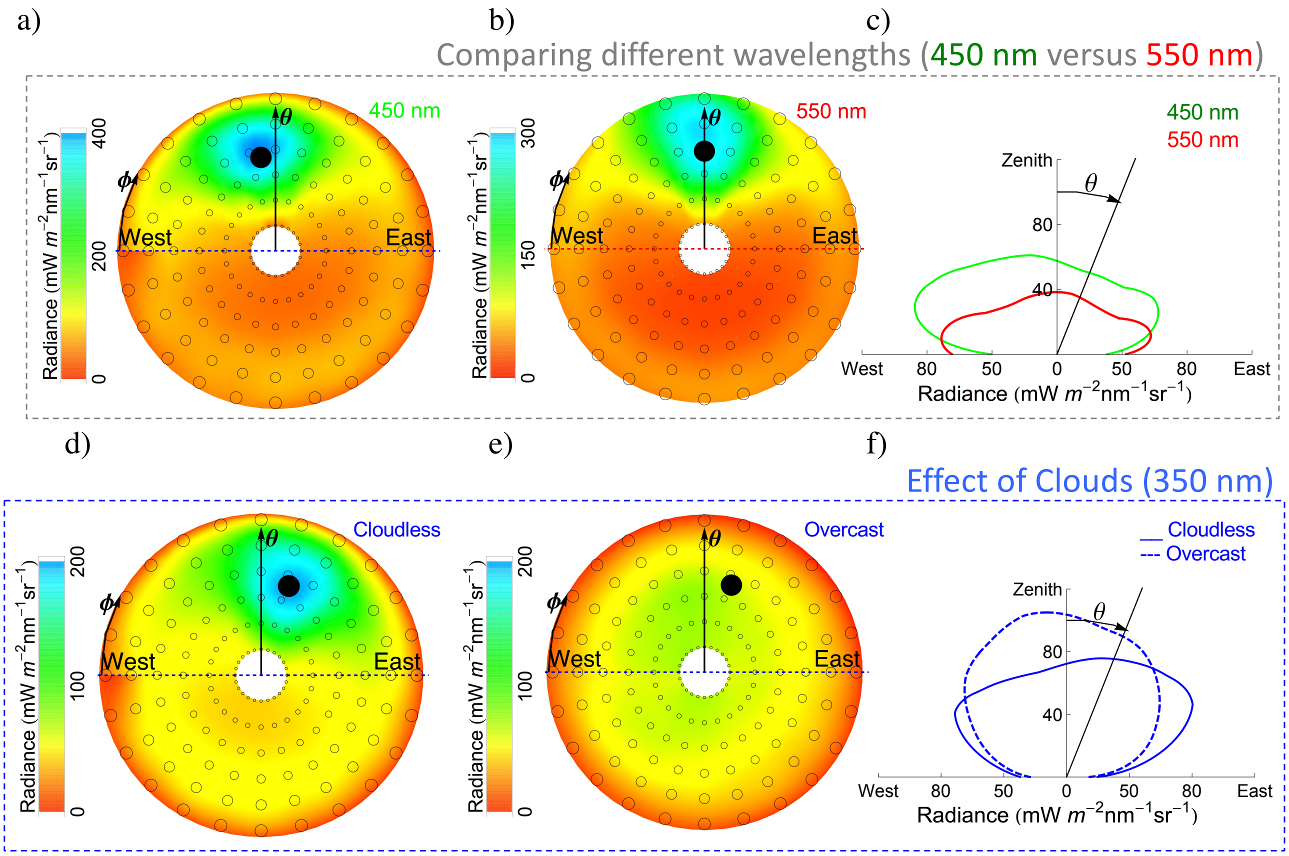

Fig. S8

Radiance distribution in Santiago (winter).

a) Radiance distribution (450 nm) measured under cloudless conditions when the AOD was 0.18. Circles indicate the measuring points except for the black filled-in circle that stands for the sun position;

b) Radiance distribution (550 nm) measured under cloudless conditions when the AOD also was 0.18;

c) Angular distribution of the radiance in the plane "West-Zenith-East". This plot shows the radiance measured changing the zenith angle (θ) while keeping the azimuth angle (ϕ) constant at 0º. Radiance data were obtained from plots a) and b).

d) Radiance distribution (350 nm) measured under cloudless conditions when the AOD was 0.2;

e) Radiance distribution (350 nm) measured under overcast conditions;

f) Angular distribution of the radiance in the plane "West-Zenith-East". Radiance data were obtained from plots d) and e). **Zenith radiance considerably increases (relative to the cloudless situation) under overcast conditions.**

Coordinates x and y in plots c) and f) were computed considering that the radiance (r) and the zenith angle (θ) are polar coordinates: x=r.cosθ y=r.sinθ. Plots were generated by using Python's Matplotlib Library [3].

.

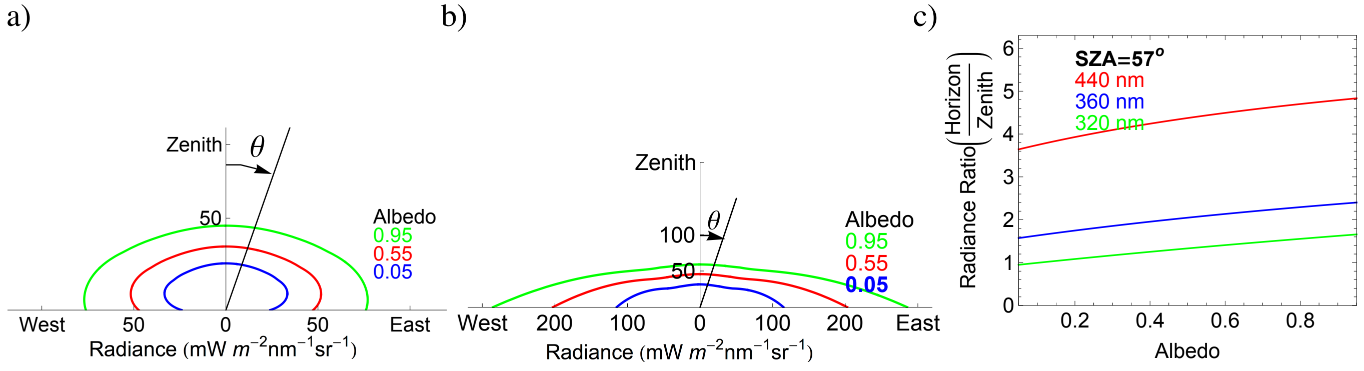

Fig. S9
Radiance distribution computed by using the UVSPEC model considering different albedos (Adapted from Cordero et al. [6]). We assumed cloudless conditions and an extremely low aerosol load (with the exception of the albedo, we used input parameters used for running the model on Union Glacier).

a) Angular distribution of the radiance (320 nm) in the plane "West-Zenith-East" (SZA=57°);

b) Angular distribution of the radiance (440 nm) in the plane "West-Zenith-East" (SZA=57°);

c) Ratio between the horizon radiance (θ=90°; ϕ=180°) and the zenith radiance (θ=0°) computed considering different albedos.

Plots were generated by using Python's Matplotlib Library [3].

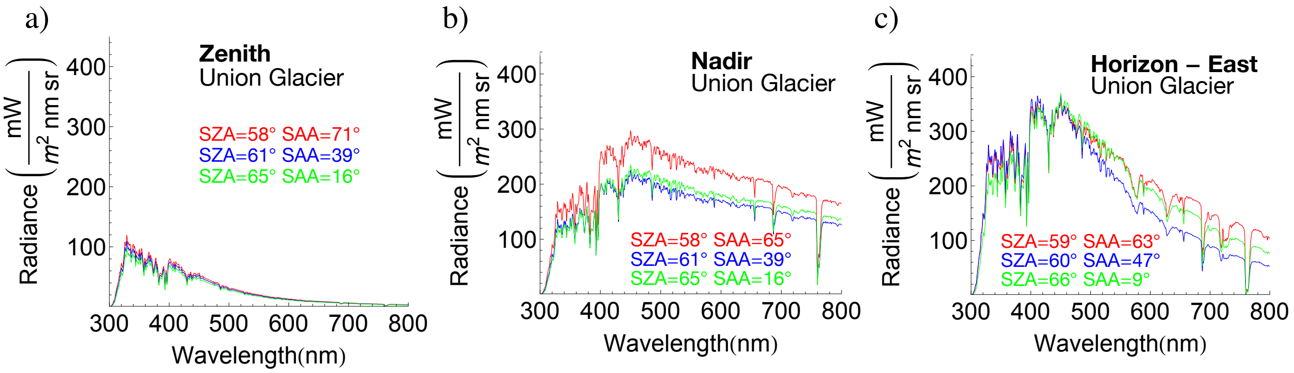

Fig. S10

Spectral measurements of the radiance on Union Glacier (early summer). The solar zenith angle (SZA) and the solar azimuth angle (SAA) at the moment of the measurements are indicated in the plots. We measured the radiance from three directions:

a) Zenith (θ=0º);
b) Nadir (θ=180º);
c) Horizon - East (θ=90º; ϕ=180º).

Plots were generated by using Python's Matplotlib Library [3].

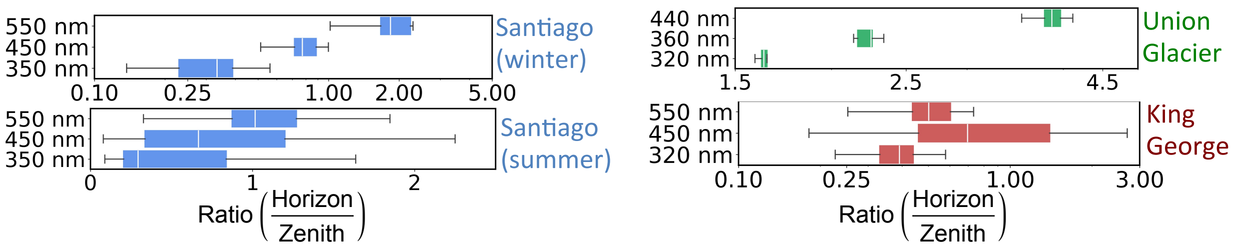

Fig. S11

Boxplots of the ratio between the horizon radiance (θ=90º; ϕ=180º) and the zenith radiance (θ=0º). In each box, the central mark (white stripe) indicates the median, and the edges indicate the 25th and 75th percentiles. The whiskers extend to the maximum and minimum data.

Plots were generated by using Python's Matplotlib Library [3].

References

1. Hersbach H. 2016. The ERA5 Atmospheric Reanalysis. AGUFM, 2016, pp.NG33D-01.

2. Platnick S, et al. The MODIS cloud optical and microphysical products: Collection 6 updates and examples from Terra and Aqua. IEEE Trans. Geosci. Remote Sens. 2016; 55(1): 502-525.

3. Hunter JD, Matplotlib: a 2D graphics environment. Comput. Sci. Eng. 9(3), 90–95 (2007). Version 3.4.3 https://matplotlib.org/stable/index.html

4. GMAO Global Modeling and Assimilation Office (GMAO) (2015), MERRA-2 tavg1_2d_rad_Nx: 2d,1-Hourly,Time-Averaged,Single-Level,Assimilation,Radiation Diagnostics V5.12.4, Greenbelt, MD, USA, Goddard Earth Sciences Data and Information Services Center (GES DISC), Accessed: May 30th, 2021

5. Cordero RR, Damiani A, Seckmeyer G, Riechelmann S, Labbe F, Laroze D, Garate F, "Satellite-derived UV Climatology at Escudero Station (Antarctic Peninsula)", Antarct. Sci. 2013; 25(6): 791–803.

6. Cordero RR, et al. Downwelling and Upwelling Radiance Distributions sampled under Cloudless Conditions in Antarctica. Appl. Opt. 2013; 52(25): 6287-94.
